# Health care cost of crusted scabies in Aboriginal communities in the Northern Territory, Australia

**DOI:** 10.1371/journal.pntd.0010288

**Published:** 2022-03-28

**Authors:** Margaret Campbell, Naomi van der Linden, Karen Gardner, Helen Dickinson, Jason Agostino, Michelle Dowden, Irene O’Meara, Meg Scolyer, Hannah Woerle, Rosalie Viney, Kees van Gool

**Affiliations:** 1 Centre for Health Economics Research and Evaluation, Faculty of Health, University of Technology Sydney, Sydney, Australia; 2 Department of Health Technology and Services Research, Technical Medical Centre, University of Twente, Enschede, the Netherlands; 3 Public Service Research Group, School of Business, UNSW Canberra, Canberra, Australia; 4 Academic Unit of General Practice, Australian National University, Canberra, Australia; 5 One Disease, Darwin, Australia; Erasmus MC, University Medical Center Rotterdam, NETHERLANDS

## Abstract

**Background:**

Crusted scabies is a debilitating dermatological condition. Although still relatively rare in the urban areas of Australia, rates of crusted scabies in remote Aboriginal communities in the Northern Territory (NT) are reported to be among the highest in the world.

**Objective:**

To estimate the health system costs associated with diagnosing, treating and managing crusted scabies.

**Methods:**

A disease pathway model was developed to identify the major phases of managing crusted scabies. In recognition of the higher resource use required to treat more severe cases, the pathway differentiates between crusted scabies severity grades. The disease pathway model was populated with data from a clinical audit of 42 crusted scabies patients diagnosed in the Top-End of Australia’s Northern Territory between July 1, 2016 and May 1, 2018. These data were combined with standard Australian unit costs to calculate the expected costs per patient over a 12-month period, as well as the overall population cost for treating crusted scabies.

**Findings:**

The expected health care cost per patient diagnosed with crusted scabies is $35,418 Australian dollars (AUD) (95% CI: $27,000 to $43,800), resulting in an overall cost of $1,558,392AUD (95% CI: $1,188,000 to $1,927,200) for managing all patients diagnosed in the Northern Territory in a given year (2018). By far, the biggest component of the health care costs falls on the hospital system.

**Discussion:**

This is the first cost-of-illness analysis for treating crusted scabies. Such analysis will be of value to policy makers and researchers by informing future evaluations of crusted scabies prevention programs and resource allocation decisions. Further research is needed on the wider costs of crusted scabies including non-financial impacts such as the loss in quality of life as well as the burden of care and loss of well-being for patients, families and communities.

## Author summary

Crusted scabies is characterised by thick skin crusting and fissuring and can lead to serious secondary infections and death from complications. Rates of crusted scabies in remote Aboriginal communities in the Northern Territory (NT) are among the highest in the world. Efforts are on the way in Australia to prevent, if not eliminate, crusted scabies. We use data from a clinical audit of 42 patients diagnosed with crusted scabies in the Top-End of the NT to estimate the health system costs associated with diagnosing, treating and managing crusted scabies for patients and their households. This cost of illness study can provide a valuable starting point for policy makers and evaluators in estimating the potential economic impact of crusted scabies prevention programs on health care service use and costs.

## Introduction

Scabies is one of the most common dermatological conditions [1–3] and is estimated to affect over 130 million people globally at any time [2,4,5]. It is caused by the *Sarcoptes scabiei* mite [6] and is commonly transmitted through direct person-to-person body contact [7]. Scabies frequently results in severe itching and may progress to “crusted (Norwegian) scabies” (CS), often in patients who have chronic conditions including Type II diabetes mellitus, chronic kidney disease, Systemic Lupus Erythematosus (SLE) and Human T-lymphotropic virus 1 (HTLV-1) which may impair their immune function [8–10].

Due to the hyper-infestation of millions of mites, individuals with CS are highly infectious to others within their household and community, and are readily able to cause significant outbreaks [8,11]. CS is a chronic, debilitating and disfiguring condition characterised by thick skin crusting and fissuring with the latter providing a pathway for the entry for bacteria, potentially causing sepsis, secondary infections, rheumatic heart disease, glomerulonephritis and death due to complications [12,13]. Individuals with CS often have poor quality of life, stigma and shame as well as repeated hospitalisations with long stays. For Aboriginal people from remote communities, CS treatment can lead to long absences from family and country [10,14–17].

Treatments for scabies and CS range from treating individual patients and their contacts to mass drug administration, treating the whole community at once, regardless of disease status [18–22]. Drugs include oral ivermectin and topical treatment options including permethrin and benzyl benzoate lotion. Elimination of scabies and CS is difficult as cured patients are susceptible to reinfestation (i.e. have recurrences) [23]. Individuals with CS are core transmitters of scabies mites and often are immunocompromised. Patients with immunosuppression are at increased risk of CS if they are infested with simple scabies. As CS is an episodic disease, guidelines recommend lifelong follow-up including regular skin checks for patients who have had CS [10,24].

Although relatively rare in the urban areas of Australia, rates of CS in remote Aboriginal communities in the Northern Territory (NT) are reported to be among the highest in the world with at least 2.4 cases per 1000 people [25]. CS became notifiable to the NT Centre for Disease Control (CDC) on 2 March 2016, under the *Notifiable Diseases Act 2016*. Risk factors for transmission of scabies and CS in the NT comprise overcrowding, a high burden of chronic disease and high levels of mobility within and between communities [9,25,26].

The aim of this study was to estimate the annual costs associated with managing and treating CS. There is currently no evidence on the resource use and costs of treating CS [27,28]. Evidence on cost of illness (COI) can inform future evaluation of CS prevention programs including cost-effectiveness analysis. This information is of value to policy makers and evaluators who wish to examine the potential impact of new prevention or management programs on health care service use, costs and health outcomes.

## Methods

### Ethics statement

Ethical approval for this study was obtained from the Human Research Ethics Committee of the NT Department of Health and Menzies School of Health Research (Ref: 2017–2940).

The perspective taken in this analysis is that of the healthcare system. Although this approach does not take into account important patient and family costs associated with CS such as lost productivity by patients, it is consistent with most economic evaluations in healthcare and is advocated by Australian guidelines on health economic evaluation and elsewhere [29]. Wherever possible, we have attempted to use the overall health system costs associated with managing CS including contributions made by third party payers (usually government) as well as out-of-pocket costs incurred by patients for prescription drugs. We have included estimates of the travel costs of patients and providers for those patients living in remote areas. In the NT, many of these costs are borne by government, and this approach is therefore consistent with our healthcare system perspective approach. The base year for all costs is 2018 and costs are reported in Australian dollars (AUD). In December 2018, one AUD was equal to 0.704 US dollars.

### Crusted scabies treatment pathway

**Fig 1:**
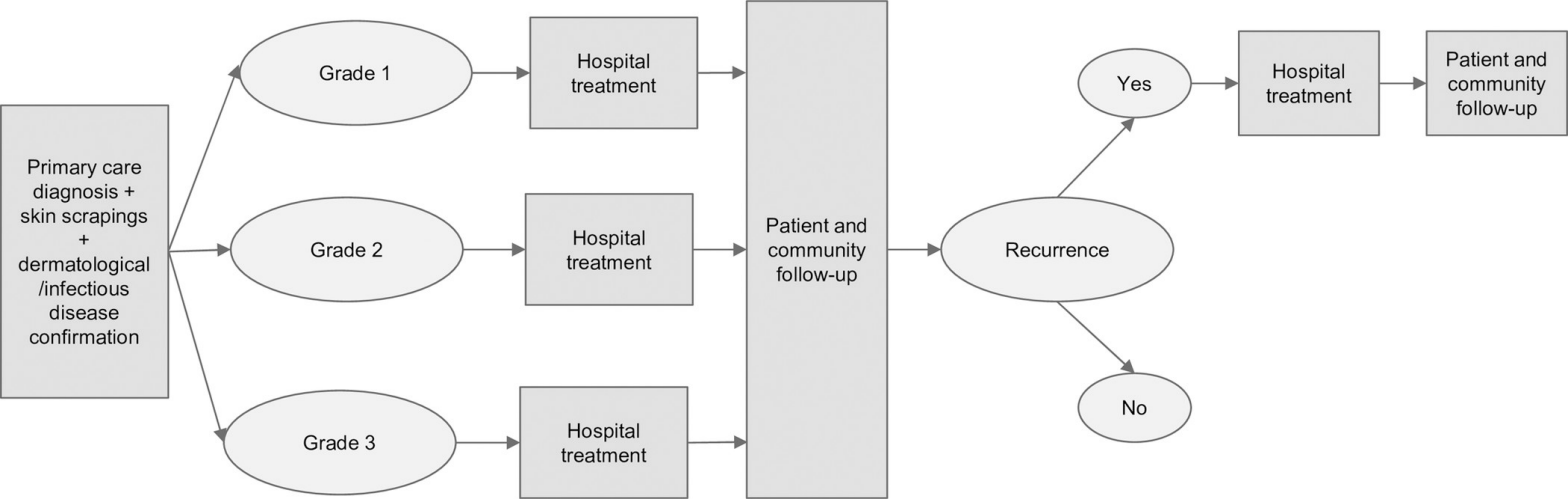
Assessment, treatment, follow-up and recurrence phases of the COI model for CS.

A treatment pathway was developed to calculate the expected average cost of treating one patient diagnosed with CS as shown in Fig 1. It covers treatment that occurs over a 12-month period from the point of diagnosis. This time horizon was selected due to the available data as well as the advice of clinical experts who suggested this timeframe would be sufficient to capture the typical maximum treatment pathway for an episode of care including potential recurrences requiring hospital re-admissions.

The entry point of the treatment pathway model is when a patient is diagnosed with CS and their disease is classified into one of three severity grades (1 = mild, 2 = moderate or 3 = severe). Severity was determined by clinical assessment across four domains: the distribution and extent of crusting; the depth of crusting; the degree of skin cracking and pyoderma; and number of previous episodes [10]. Costs associated with the diagnosis of CS include the initial clinic visit (incorporating collection of blood samples and skin scrapings of suspected crusting or mite burrows), diagnostic tests for *Sarcoptes scabiei* mites, chronic kidney disease (CKD), Type II diabetes mellitus, SLE and HTLV-1 as well as specialist review and health checks associated with the diagnosis of CS [30].

Following diagnosis, the patient moves into a grade-dependent treatment phase which is provided in hospital and in many instances involves an emergency department (ED) presentation. ED presentations are a common way for patients to be admitted to public hospitals. Once the acute phase of treatment is complete, the patient moves into a follow-up phase involving not only the patient but also their family and other household members to make the household a ‘scabies-free zone’ to reduce the risk of reinfestation of the patient and infestation of others prior to patients returning home from hospital [10,24]. This includes washing potentially contaminated clothes and bed linen at high temperature, treatment of the family and other household members and funding of household and community support initiatives [10,24,31].

The treatment pathway allows for the probability of CS recurrence to vary with the grade of the initial episode. In this paper, recurrence is a confirmed CS diagnosis occurring more than three months after the initial CS episode, where treatment was completed. This period was chosen in an attempt to reflect this was a new CS infestation after the patient returned home, rather than a reactivation of an inadequately treated initial infestation [32]. Fig 1 illustrates the assessment, treatment, follow-up and recurrence phases of the COI model for CS.

Populating the treatment pathway requires four key data inputs:

1. Incidence of CS by severity grade. This refers to the likelihood of being diagnosed with grade 1, 2 or 3 CS.
2. Healthcare resource use associated with the diagnosis, treatment and/or follow-up of CS.
3. Unit costs to quantify the value of each of the resource use items.
4. The probability of a patient suffering a recurrence of CS conditional on grade.

The expected cost of treating one CS patient over a twelve-month period is set out in Eq 1:

$$E_{i}=\bar{D}+\sum_{s=1}^{3}{(P}_{s}*C_{s})+\bar{F}+\sum_{s=1}^{3}{(R}_{s}*C_{1})$$ (1)

Where the expected cost E_i_ for each patient (i), is $\bar{D}$ plus $\bar{F}$, which are the average cost per patient associated with the diagnosis and follow-up of CS, respectively. P_s_ is the probability of being diagnosed with severity grade, s, and C_s_ is the associated acute treatment cost for each severity grade, s. R_s_ is the likelihood of recurrence conditional on the severity grade (s) of the initial CS episode. The costs associated with recurrence (C) are based on the treatment and follow-up costs of an episode with severity grade 1 (s = 1). The overall cost of CS for the population can then be calculated by multiplying E by the incidence of CS over a given period of time.

### Data collection

A manual audit of NT’s Top-End shared electronic health records was undertaken. The Top-End covers an area of 245,000 square kilometres and incorporates the city of Darwin as well as Arnhem Land and the Katherine region. There are 25 Aboriginal Community Controlled Health Clinics and over 30 NT Government clinics in the Top-End providing services to a total population of 200,000 people. These audits were conducted as part of continuous quality improvement activities by *One Disease* (OD) staff to provide health services with information on CS clients to improve CS care [31]. *One Disease* is a philanthropically funded organisation that aims to eliminate CS from the NT (see https://www.onedisease.org/ for more details).

The *One Disease* staff identified the cohort by selecting people from Top-End communities participating in the *One Disease* program that had been notified with CS and had opted into the shared electronic health record (SEHR). The SEHR records contain information from all places where patients receive healthcare. To verify the identification of the audit cohort, their demographic information was matched to the CDC CS notifications dataset. All except one audit record was matched.

Patients were included in the audit if they had an episode of CS between July 1, 2016 and May 1, 2018. Here, diagnosis of CS is based on specialist assessment in accordance with the Central Australian Rural Practitioners Association (CARPA) guidelines [24]. *One Disease* staff determined the severity grade based on information contained in the electronic health records.

To identify health care use related to the treatment and management of CS, the CS management protocol from the CARPA Standard Treatment Manual [24] was used to facilitate decisions on which health care resource use variables to extract from the electronic health record as part of the audit. These variables included the number and type of clinical visits, pathology tests, hospitalisations, pharmaceuticals and community follow-up activities.

### Unit costs

#### Initial assessment phase

Table 1 shows the unit costs for items associated with the initial assessment of the treatment pathway. The types of resources required to diagnose and arrange treatment for patients were identified through the audit. Unit cost information was derived from the Medicare Benefits Schedule (MBS) for medical consultations and diagnostic test costs [33] and the Pharmaceutical Benefits Scheme (PBS) for the prescription drug costs [34]. Where required, these sources were supplemented with information from the CARPA Standard Treatment Manual [24] and an online Pharmacy company for medicines and treatments that are not listed on the PBS [35]. Input from *One Disease* staff was also sought regarding the unit cost of the HTLV-1 test and patient travel to the hospital [31].

**Table 1:** Unit costs for resources used during the initial assessment of CS.

Description	Unit	Unit cost ($)
Blood glucose test–HbA1c^a^	test	14.30
Urea, electrolytes, creatinine, microalbumin- quantitation in urine & full blood count for chronic kidney disease^a^	test	60.90
Antinuclear antibodies; antibody to double-stranded deoxyribonucleic acid, full blood count for SLE^a^	test	43.35
Screening assay and analysis at pathology laboratory for HTLV-1^b^	test	40.00
1^st^ assessment for CS at the patients’ primary health care service^b^	visit	212.25
Skin–microscopy & culture of material from microscopy and culture of superficial sites^a^	test	28.70
Full blood count^a^	test	14.45
Liver function test & C-Reactive Protein^a^	test	29.50
Human Immunodeficiency Virus^a^	test	13.35
T-cell subsets^a^	test	88.70
Return plane charter for remote locations outside Darwin^b^	Travel	2000.00
Return plane charter for very remote locations outside Darwin^b^	Travel	5000.00

Sources: a = MBS [33]

b = OD [31]

#### Hospital phase

The audit revealed hospital stays for patients diagnosed with CS were classified under a range of Australian Refined Diagnostic Related Groups (AR-DRG). There is no specific AR-DRG for the treatment of crusted scabies. Instead, most CS patients (66%) were classified under AR-DRGs that relate to the treatment of skin-related and infectious diseases. For the remaining CS patients, their hospitalisations were classified under AR-DRGs that primarily relate to their co-morbidity. In each hospital record, regardless of which AR-DRG was used, CS was listed as a diagnosis and reason for the hospitalisation.

The AR-DRGs identified in the audit provide the basis for estimating hospital costs. The costs reported in the 2018/19 National Hospital Cost Data Collection (NHCDC) [36] was matched to each patient’s AR-DRGs code as identified in the audit. We excluded overheads and only used direct costs in this analysis.

Comparing the length of stay (LOS) for patients in our audit with those in the NHCDC revealed considerable differences. For example, the national average LOS for a common skin-related hospital stay (AR-DRG J68A) is 5.1 days, whereas patients in our audit, under the same AR-DRG, had an average LOS of 11.6 days. The longer LOS for patients in our sample may reflect the nature of treating CS compared to other skin conditions that are classified under the relevant AR-DRGs. This also implies that the costs of treating a patient with CS may be higher than the national average cost reported in the NHCDC. Hence, relying on the national average could systematically underestimate the true cost of treating a hospital CS episode. For this reason, we have adjusted the hospital cost to estimate the additional resource use associated with treating CS.

The method for adjusting hospital costs involved identifying the cost for each extra day of hospital stay by estimating a *daily add-on cost*. To do this, we used the NHCDC to isolate hospital costs that tend to occur towards the end of a hospital stay (e.g., medical ward, nursing costs and allied health) from the costs that occur at the start of a hospital admission (e.g., costs associated with critical care, operating rooms, emergency departments and special procedure suites). We then divide this cost by the national average LOS for each relevant AR-DRG to arrive at a daily add-on cost estimate. This daily add-on cost varies by AR-DRG but on average was $1,112.

The second step in our hospital cost adjustment method was to identify the excess LOS for each episode. For each patient in our sample, we calculated the difference between their LOS and the national average for that particular AR-DRG. Eq 2 shows the adjusted hospital cost calculation per hospital episode:

$${Adj\;Cost}_{i}={Dir\;Cost}_{drg}+({ALOS}_{drg}-{LOS}_{i})*{DailyAdd\;On}_{drg}$$ (2)

Where the adjusted hospital cost *(Adj Cost*_*i*_*)* estimated for each patient, *i*. The direct costs, *Dir Cost*_*drg*_, is reported through the NHCDC for each relevant AR-DRG. The excess LOS, as defined above, is multiplied by the daily add-on cost, *DailyAddOn*_*drg*_, that is specific to each AR-DRG. The mean of the adjusted costs (*Adj Cost*_*i*_) for each severity grade are then calculated and applied to Eq 1, above.

#### Community follow-up phase

Table 2 reports the unit costs for the community-follow up that covers both patient and household care. This phase comprises the following items: contact tracing, CS recall if a patient with crusted scabies does not self-present to the clinic; patient clinic visit and medications; treatment of the household prior to the patient’s hospital discharge. In some instances, this involves travel costs for the health or community workers.

**Table 2:** Unit costs for resources used during the community follow-up phase of CS.

	Description	Unit	Unit cost $
Patient	Services provided by a practice nurse or Aboriginal and Torres Strait Islander health practitioner on behalf of a medical practitioner^a^	Clinic visit	24.00
	CS recall: salary remote area nurse N4: $47.66/hr^c,^ + driver $28.77/hr (both including 30% on-costs)^b^	Phone or visit	105.00
	Ivermectin (3mg tablet, 4 tablets in pack; 1 dose = 4 tablets)^d^	Dose	46.49
	Permethrin (5% cream, 30 g) ^d^	Dose	18.64
	Calmurid (10% urea, 5% lactic acid in moisturising cream)^e^	Dose	2.67
	1st stage of disease notification and contact tracing requires eight hours of time: Salary remote area nurse N4: $47.66/hr (including 30% on-costs) ^b,c^	8 hours	381.00
Household	Treatment of household: requires salary remote area nurse N4 and driver for 24 hours $47.66/hr and $28.77/hr, respectively (includes 30% on-costs)^b,c^	hours	1834.00
	Permethrin 5% (Lyclear), 30g (20 tubes per household treatment)^d,f^	Dose	18.64
	Ivermectin (4 tablets = 1 dose = 1 packet)^d^	Dose	46.49
	Travel to remote location (0.68/km * 400km round trip)^g^	Travel	272.00

Sources: a = MBS[33]

b = OD[31]

c = Northern Territory Public sector Nurses and Midwives 2014–2018 Enterprise Agreement

d = PBS[34]

e = Pharmacy Direct[35]

f = CARPA Standard Treatment Manual[24]

g = Australian Taxation Office (ATO) [37]

A number of assumptions had to be made to estimate some unit costs. We assumed all patients fall into 60–70 kilograms weight bracket to calculate the required doses of medications. This weight range implies that four tablets (1 pack) of ivermectin was required. Further, we assumed that approximately 30 g/ per application was required for Permethrin; and Calmurid required 25g per application per person and that there were four applications to each tube. Finally, the household follow-up was estimated to take 24 hours over a one-week period and required a registered nurse and driver. This 24-hour period includes an average of eight hours travel time for each staff member. We estimated that for those patients living in remote areas, the round trip is approximately 400km from the treating clinic to the household location.

## Results

The manual audit of NT’s shared electronic health records identified 42 patients diagnosed and treated for CS over the observation period. Table 3 reports a range of baseline patient characteristics. The median and mean age of the cohort were 47 and 49, respectively. All patients identified as having an Aboriginal or Torres Strait Islander background and the majority of patients were female (67%). Darwin was the most common place of residence (43%) whereas 29% of the cohort lived in remote areas and the same percentage lived in very remote areas. This compares to 76.6% of the overall Aboriginal community in the Northern Territory who reside in remote or very remote areas (NT Government). Of the 42 patients identified in the audit, 41 had data on the grade of their initial episode. There were even numbers of patients diagnosed with Grade 1 and 2 CS and a smaller proportion was diagnosed with Grade 3 CS.

**Table 3:** Characteristics of individuals with episode of crusted scabies between July 2016—May 2018, Top End Northern Territory.

Characteristic	One Disease audit
Individuals (n)	42
Median/mean age (years)	47/49
Age range (years)	21–71
Sex	28 (67%)
• Female	
• Male	14 (33%)
Aboriginal or Torres Strait Islander background	42 (100%)
Location	
• Urban/Metropolitan (Darwin)	18 (43%)
• Remote	12 (29%)
• Very remote	12 (29%)
Grade for initial episodes • Grade 1	15 (36%)
• Grade 2	15 (36%)
• Grade 3	11 (26%)
• Grade missing	1 (2%)

### Initial assessment phase resource use and costs

The expected costs per patient (average number of units times unit costs) are shown in Table 4 for each health care product or service utilized during the initial assessment phase. All resource use data was derived from the OD audit. The initial phase largely consists of tests and primary care consultations as well as travel to hospital for those patients residing in remote and very remote communities to commence their treatment. The overall expected cost of the initial phase is $2,450 per patient diagnosed with CS.

**Table 4:** Resource use and expected cost of the CS initial assessment phase.

Description	Unit	Mean units per patient	Expected cost ($)	Std. dev ($)
Blood glucose test—HbA1c	test	0.93	13.30	3.73
Urea, electrolytes, creatinine, microalbumin- quantitation in urine & full blood count for chronic kidney disease	test	0.95	58.00	13.13
Antinuclear antibodies; antibody to double-stranded deoxyribonucleic acid, full blood count for SLE	test	0.55	23.74	21.83
Screening assay and analysis at pathology laboratory for HTLV-1	test	0.76	30.48	17.24
1st assessment for CS at the patients’ primary health care service	visit	1	212.25	0
Skin—microscopy & culture of material from microscopy and culture of superficial sites	test	0.98	28.02	4.43
Full blood count	test	0.90	13.07	4.29
Liver function test & C-Reactive Protein	test	0.90	26.69	8.76
Human Immunodeficiency Virus	test	0.76	10.17	7.76
T-cell subsets	test	0.38	33.79	43.60
Return plane charter for remote locations outside Darwin	Travel	0.29	571.43	2095.00
Return plane charter for very remote locations outside Darwin	Travel	0.29	1428.57	2095.00
Total initial assessment phase			2450.00	2093.00

### Adjusted hospital cost

Table 5 presents a summary of the mean direct hospital costs and average length of stay as reported in the NHCDC by severity grade. This is based on a sample of 40 initial episodes of CS out of a total of 42 patients in our sample. One patient was lost from the sample due to lack of grade information and one patient was lost due to lack of DRG information. Table 5 also reports the average LOS for CS patients as identified in the audit, the daily add-on costs and the adjusted cost by grade. The table shows that CS patients have longer LOS than the national average—particularly for patients diagnosed with severity grade 2 or 3. The daily add-on cost is fairly consistent regardless of grade but due to the higher excess LOS for grades 2 and 3, the adjusted costs for these grades are higher. There is only weak evidence (p = 0.0983) that the mean cost for grade 1 is different to the mean cost for patients with grade 2 or 3. There is no statistical difference between the mean cost of grades 2 and 3.

**Table 5:** Average hospital costs, length of stay and adjusted cost per episode, by severity grade.

	National Hospital Costs Data Collection		CS LOS	Excess LOS	Daily add-on cost	Adjusted cost Mean
Severity grade	Direct costs $	LOS Days	Days	Days	$	$
1 (n = 14)	12,673	7.81	11.33	4.33	1,058	16,874
2 (n = 15)	10,565	6.26	19.07	12.81	1,133	24,612
3 (n = 11)	13,070	7.97	19	11.03	1,151	25,794
Weighted average	11,992	7.27	16.22	9.35	1,112	22,229

**Fig 2:**
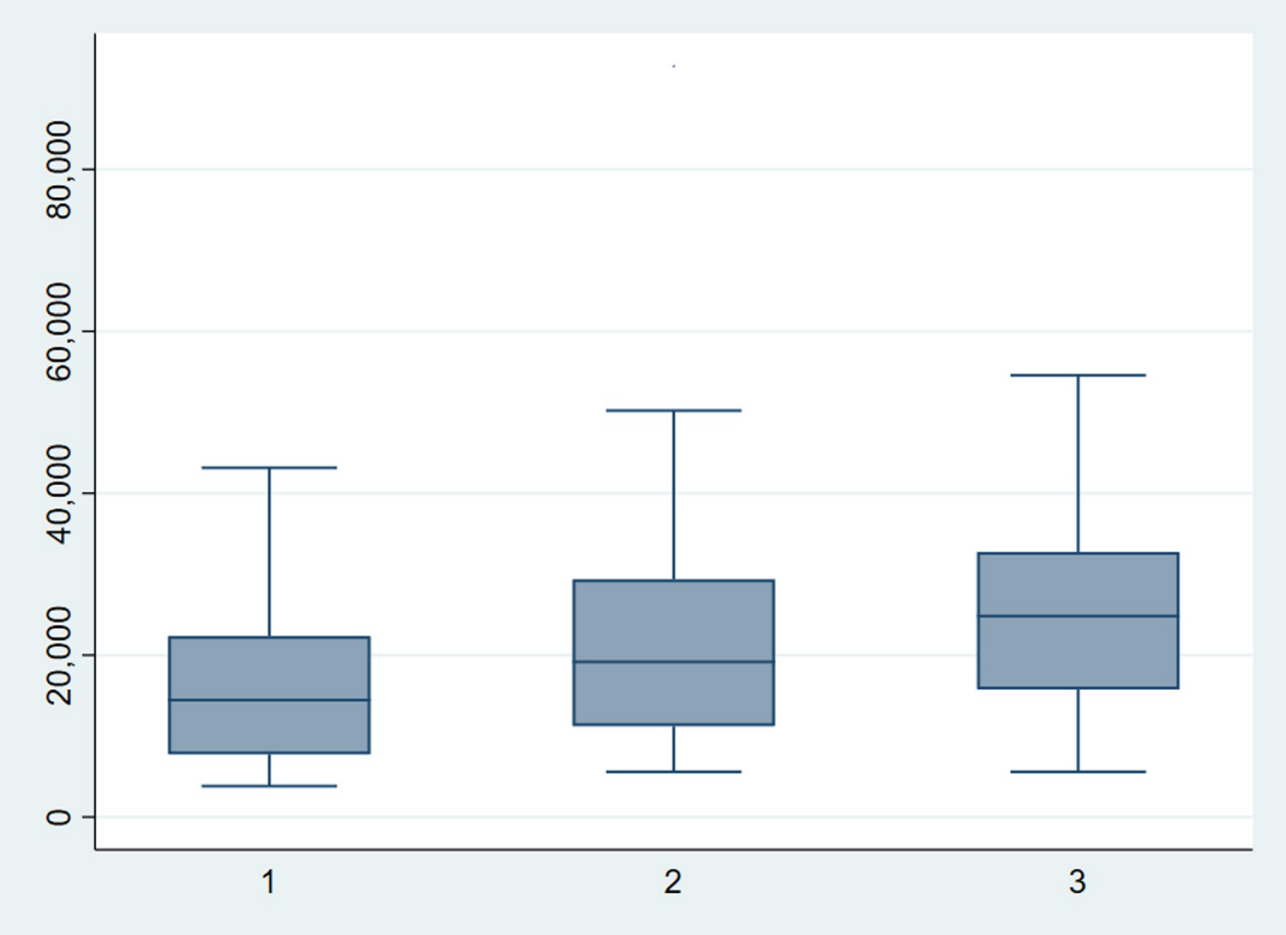
Box plot of adjusted hospital costs by severity grade.

Fig 2 illustrates the distribution of adjusted hospital costs by CS severity grade. For grades 1, 2 and 3 the median is $14,450, $19,164 and $24,824, respectively. For each grade, the median is slightly less than the mean reported in Table 5, suggesting that the cost distribution is skewed. The interquartile range for severity grade 1 adjusted hospital costs is $14,592; for grade 2 it is $18,111; and for grade 3 it is $16,979.

### Probability of recurrence and associated hospital costs

The probability of recurrence was derived, using the clinical audit data. To estimate the recurrence rate, notifications with grade information and at least 12 months’ worth of subsequent observation time were categorised as an initial CS episode. This brought the initial episode count down to 35 and these were counted as the denominator for calculating recurrences. Any notification that occurred within a 12-month period of an initial notification for any specific patient was counted in the numerator.

Through this categorisation process, nine patients had a total number of thirteen recurrent episodes. For all recurrent episodes we identified the grade of the preceding initial episode. This enabled us to estimate the probability of recurrence by grade. Table 6 shows there is a fairly even number of patients in each initial severity grade but the point estimates indicate the chances of recurrence were higher for patients diagnosed with greater severity in their *initial* episode. For example, 45% of patients with an initial severity grade of 3 go on to have a recurrence within 12 months. The corresponding figure patients with severity grade 1 is 27%. As shown in Table 6 due to the very low sample size, the confidence intervals are wide.

**Table 6:** Probability of recurrence within a 12-month period by initial severity grade.

Initial severity grade			Recurrence			
Grade	Episodes	Proportion	Episodes	Proportion	95% Confidence Interval	
					Lower bound	Upper bound
1	11	0.31	3	0.27	0.01	0.54
2	13	0.37	5	0.38	0.12	0.65
3	11	0.31	5	0.45	0.16	0.75
Overall	35	1.00	13	0.37	0.21	0.53

For the very small number of recurrent episodes (n = 13), grade was poorly reported. For those with grade information available, grade 1 was the most common severity score. The hospital cost for recurrent episodes were therefore deemed to be equivalent to the cost of a grade 1 episode (i.e., $16,874).

### Follow-up phase resource use and costs

Table 7 presents the resource use and expected costs of the follow-up phase of care, including resources used for the treatment of the household prior to the patient’s return to their community. Upon discharge from hospital, patients will have been treated and have a clear skin scraping. As a result, we do not expect post-hospital treatment cost to be influenced by the initial grade of CS severity.

The most resource intensive aspect of the community follow up are the clinical visits provided by a practice nurse or Aboriginal or Torres Strait Islander health practitioner. Overall, each patient’s follow-up costs were estimated to be $3,261. This amount was also used as the basis for estimating the follow-up costs for patients who experienced a recurrent episode.

**Table 7:** Resource use and expected cost of the CS patient and community follow-up phase.

		Unit	Resource use	Expected cost ($)	Std. dev ($)
Patient treatment	CS recall: salary remote area nurse N4: $47.66/hr^,^ + driver $28.77/hr (both including 30% on-costs)	Phone call or visit	0.54	50.00	53.10
	Services provided by a practice nurse or Aboriginal and Torres Strait Islander health practitioner on behalf of a medical practitioner	Clinic visit	66.26	1590.24	2279.20
	Ivermectin (3mg tablet, 4 tablets in pack; 1 dose = 4 tablets)	Dose	2.21	102.74	185.26
	Permethrin (5% cream, 30 g)	Dose	5.83	108.73	238.35
	Calmurid (10% urea, 5% lactic acid in moisturising cream)	Dose	5.4	14.43	41.04
	1st stage of disease notification and contact tracing requires eight hours of time: Salary remote area nurse N4: $47.66/hr (including 30% on-costs)	hours	8	381.28	0.00
Household treatment:	Treatment of household: requires salary remote area nurse N4 and driver for 24 hours $47.66/hr and $28.77/hr, respectively (includes 30% on-costs)	household treatment	0.38	698.67	901.42
	Permethrin 5% (Lyclear), 30g (20 tubes per household treatment)	Dose	7.6	142.02	183.23
	Ivermectin (4 tablets = 1 dose = 1 packet)	Dose	0.38	16.67	22.85
	Travel to remote location (0.68/km * 400km round trip)	Travel	0.54	155.43	136.24
	Expected follow-up costs			3261.22	2567.95

### Summary of expected cost per patient and population

The expected cost per patient for each of the three phases of the crusted scabies model was calculated by multiplying the average number of units used for each resource item by its unit cost and summing these values. As shown in Table 8, over a one-year period, the total expected cost for treating one CS episode is estimated to be $35,418. The associated 95% confidence interval of the mean cost is $27,000 to $43,800. In 2017, 44 patients were diagnosed with CS in the Northern Territory [9]. Thus, the total annual cost for managing CS in the Northern territory is $1,558,392 with a 95% confidence interval of $1,188,000 to $1,927,200. Conversely, this amount would be the anticipated annual health system savings if CS can be eliminated.

**Table 8:** Expected cost for each of the components of the model for patients with crusted scabies.

Model phase	Expected cost per patient ($)	Standard error of mean
Initial assessment	2,450	323
Hospital	22,229	2709
Patient and community follow-up	3,261	396
Recurrence costs		
Hospitalisation	6,267	2085
Follow-up	1,211	402
Total health system cost per episode	**35,418**	**4285**

Alternative objectives to full elimination are to reduce the grade severity of CS at the point of diagnosis as well as the number of recurrences. Such objectives can also lead to substantive health system savings. If, for example, all CS episodes were classified as grade 1, the overall health system savings would amount to $235,670. If, on the other hand, all recurrences can be eliminated, overall health system savings would amount to $329,032. Meeting the twin objectives of eliminating higher grades CS and recurrences would therefore lead to an expected savings of $564,702 per year.

## Discussion

This paper provides the first analysis of the cost of treating CS. Using data extracted from the electronic record delivered a rich source of information on CS diagnosis, treatment and follow-up care. Based on the data, we developed a treatment pathway of CS that accounts for the probability of recurrence and resources used to diagnose, treat and follow-up patients with CS. Furthermore, the model differentiates treatment costs based on the severity grade at the point of diagnosis.

The expected health care cost per patient diagnosed with CS is $35,418 resulting in an overall cost of $1,558,392 for managing all patients diagnosed in the Northern Territory in a given year (2018). By far, the biggest component of the health care costs falls on the public hospital system that are funded by Australia’s federal and state governments. Although patient out-of-pocket costs are a common feature of Australia’s health care system, in the treatment of CS such costs are minor. This is because most primary care services offered to the Aboriginal community are free at the point of service and all public hospital services are offered free of charge.

The cost of CS to the patient and community, however, goes well beyond the treatment required. Future research should focus on the burden of CS beyond health system costs, including productivity costs, loss in quality-of-life to the patient and the household. For Aboriginal people from remote communities, CS may also lead to a loss of well-being associated with long absences from family and country. Future models of the disease pathway should also incorporate the potential impact of CS on household members and, perhaps, even local communities. By elaborating on this aspect of the model, it will become feasible to undertake future analysis of elimination/prevention programs and their wider impact on not just patients but also households and communities.

Although our sample size is small, our audit captures around 55% of patients diagnosed with CS in the NT over the observation period [9]. Further, our sample has a very similar distribution of severity grades compared to those in NT CDC data [9]. This provides some re-assurance that our sample is representative of the population for this important model input.

Our analysis has some limitations. First, whilst we attempted to isolate the health care costs of treating CS from other underlying diseases, it is plausible that some aspects of a patient’s hospitalisation also captures the cost of treating other conditions. This potentially exaggerates the CS costs. However, it should be noted that only those hospital episodes where CS was being treated were included in the analysis. As CS may have complicated the management of other underlying conditions, it is appropriate that such costs are included. Another limitation is that our analysis was based on CS treatment patterns observed between 2016 and 2018. Treatment patterns may evolve and therefore change the cost of illness. This is why we have reported resource use items and unit costs in detail, so that future evaluators can take such potential practice changes into account and substitute new patterns of care and associated costs into the model.

Finally, one of the limitations of our approach is that we assumed the timeframe for an episode of care lasts (at most) 12 months. When we examined the data, this timeframe was deemed appropriate because over the entire 22-month observation period, there were no additional recurrences (i.e., hospitalisations) for any patients after 12 months.

Notwithstanding these limitations, our analysis provides a basis for future cost-effectiveness analysis on the impact of CS prevention programs. In addition, this COI analysis can also be used to examine the impact of the program on preventing higher grades of CS through earlier recognition and treatment, as well as efforts to reduce the incidence of recurrence.
